# Crosstalk between Non-Coding RNAs and Wnt/β-Catenin Signaling in Head and Neck Cancer: Identification of Novel Biomarkers and Therapeutic Agents

**DOI:** 10.3390/ncrna9050063

**Published:** 2023-10-17

**Authors:** Anjana Sajeev, Bandari BharathwajChetty, Ravichandran Vishwa, Mohammed S. Alqahtani, Mohamed Abbas, Gautam Sethi, Ajaikumar B. Kunnumakkara

**Affiliations:** 1 Cancer Biology Laboratory, Department of Biosciences and Bioengineering, Indian Institute of Technology (IIT) Guwahati, Guwahati 781039, Assam, India; s.anjana@iitg.ac.in (A.S.); bharathwajn@iitg.ac.in (B.B.); v.vishwa@iitg.ac.in (R.V.); 2 Radiological Sciences Department, College of Applied Medical Sciences, King Khalid University, Abha 61421, Saudi Arabia; mosalqhtani@kku.edu.sa; 3 BioImaging Unit, Space Research Centre, Michael Atiyah Building, University of Leicester, Leicester LE1 7RH, UK; 4 Electrical Engineering Department, College of Engineering, King Khalid University, Abha 61421, Saudi Arabia; mabas@kku.edu.sa; 5 Department of Pharmacology, Yong Loo Lin School of Medicine, National University of Singapore, Singapore 117600, Singapore

**Keywords:** head and neck cancer, non-coding RNAs, Wnt/β-catenin signaling, anti-miR

## Abstract

Head and neck cancers (HNC) encompass a broad spectrum of neoplastic disorders characterized by significant morbidity and mortality. While contemporary therapeutic interventions offer promise, challenges persist due to tumor recurrence and metastasis. Central to HNC pathogenesis is the aberration in numerous signaling cascades. Prominently, the Wnt signaling pathway has been critically implicated in the etiology of HNC, as supported by a plethora of research. Equally important, variations in the expression of non-coding RNAs (ncRNAs) have been identified to modulate key cancer phenotypes such as cellular proliferation, epithelial-mesenchymal transition, metastatic potential, recurrence, and treatment resistance. This review aims to provide an exhaustive insight into the multifaceted influence of ncRNAs on HNC, with specific emphasis on their interactions with the Wnt/β-catenin (WBC) signaling axis. We further delineate the effect of ncRNAs in either exacerbating or attenuating HNC progression via interference with WBC signaling. An overview of the mechanisms underlying the interplay between ncRNAs and WBC signaling is also presented. In addition, we described the potential of various ncRNAs in enhancing the efficacy of chemotherapeutic and radiotherapeutic modalities. In summary, this assessment posits the potential of ncRNAs as therapeutic agents targeting the WBC signaling pathway in HNC management.

## 1. Introduction

Head and neck cancers (HNC), encompassing malignancies arising from the oral cavity, oropharynx, nasopharynx, hypopharynx, and larynx, represent a significant global health burden, as underscored by their pronounced prevalence [1]. The high prevalence of squamous cell carcinoma (SCC) among HNCs (about 95%) led to the belief that it is a relatively uniform disease when compared to other types of cancers [1]. However, emerging evidence delineates the notable heterogeneity inherent to head and neck squamous cell carcinoma (HNSCC), which hinders the accurate prognostication, therapeutic strategy formulation, and, from a molecular perspective, identification of key oncogenic determinants [1,2,3]. Established risk factors for HNC include tobacco consumption, excessive alcohol intake, and infection with the human papillomavirus (HPV), with the disease accounting for over about 444,347 mortalities in the year 2020 [4,5,6,7]. Contemporary therapeutic modalities for HNC include concurrent chemoradiation, chemotherapy, radiotherapy, and surgical interventions. Nonetheless, the persistence of suboptimal patient survival rates and adverse sequelae associated with these interventions underlines the therapeutic challenges [5,8,9,10]. Consequently, the elucidation and development of innovative therapeutic targets, as well as compounds with anti-neoplastic, anti-metastatic, and anti-angiogenic properties that interact with salient proteins or signaling pathways pivotal to tumor progression, are of critical significance for cancer therapy [2,11,12,13,14,15,16,17].

One of the core signaling processes responsible for regulating cell proliferation, cell polarity, and cell fate determination in embryonic development and tissue homeostasis is signaling via the Wnt family of secreted glycolipoproteins [18,19,20,21]. Wnt signaling consists of two interconnected and mutually regulated signaling cascades, canonical and non-canonical pathways. Canonical Wnt signaling, or the Wnt/β-catenin (WBC) pathway, is dependent on β-catenin translocation into the nucleus and the subsequent transcription of target genes [22]. Contrarily, the non-canonical pathway operates independently of β-catenin and its affiliated transcription factors, including T-cell factor/lymphoid enhancer-binding factor (TCF/LEF). While the canonical axis predominantly influences cellular proliferation, the non-canonical circuitry primarily oversees cellular polarity and migration [22]. Additionally, the Wnt signaling pathway in humans comprises 19 different Wnt proteins that are evolutionarily conserved cysteine-rich glycoproteins and are integral for the self-renewal process in many mammalian tissues [23,24,25]. Beyond these roles, Wnt signaling extends to encompass hepatic differentiation, pulmonary tissue regeneration and repair, hair follicle renewal, hematopoietic system development, organ aging, and osteoblast maturation [24,25,26,27].

In recent years, the emergence of non-coding RNA (ncRNA) has profoundly expanded our understanding of cellular and molecular biology. Intriguingly, based on Ensemble1 (v76) data, a mere 34% of the human transcriptome encodes proteins, while the residual 66% encompasses non-coding genes. This segment includes entities such as ncRNAs, antisense RNAs, and pseudogenes, among others [28,29,30]. Cumulative research over the past decades has delineated the pivotal role of ncRNAs in modulating transcription across various strata [31]. Hence, these RNAs have emerged as master regulators of gene expression, notwithstanding their non-protein-coding nature. For example, a recent investigation examined the expression patterns of three microRNAs (miRNAs), a prominent subclass of ncRNAs, in laryngeal neuroendocrine carcinoma (LNEC), an infrequent subtype of HNC. The study demonstrated a pronounced downregulation of miR-133b in LNEC patients, positioning it as a tumor suppressor. In contrast, miR-223 and miR-449a manifested oncogenic properties [32]. Considering the clinical challenge posed by metastasis of laryngeal cancer (LC) cells to cervical lymph nodes, targeted therapeutic strategies are imperative. A noteworthy investigation revealed that the ectopic expression of miR-449a curtailed both proliferation and invasiveness of LC cells, concurrently downregulating Notch1 and Notch2, thereby positioning miRNAs as prospective therapeutic markers for nodal metastasis in LC [33]. In light of these findings, this review endeavors to furnish an exhaustive analysis of the interplay between ncRNAs and proteins within signaling cascades, emphasizing their influence on the WBC pathway and its mechanistic ramifications in HNC.

## 2. Wnt Signaling and Cancer

The components of the WBC signaling pathway were initially elucidated through genetic investigations in Drosophila [34]. In 1982, Nusse and Varmus identified the mouse Wnt1 gene, originally termed Int-1, as a favored integration locus for the mouse mammary tumor virus (MMTV) in the induction of mammary carcinomas [35]. A few years later, scientists unraveled the link between mutations in the adenomatous polyposis coli (APC) gene and the progression of hereditary colon cancer, which strongly cemented the close association between Wnt signaling and colorectal cancer [36]. Later, many studies showcased the importance of this pathway in the initiation, development, and progression of different cancers.

The WBC signaling pathway operates through a complex network of interactions and signaling events. At the center of this pathway is β-catenin, a protein that plays a dual role as a core component of the pathway and a transcriptional co-factor [24]. In the absence of Wnt ligands, cytoplasmic β-catenin is constantly targeted for degradation through a series of phosphorylation events orchestrated by the Axin complex, which is comprised of scaffolding protein, Axin, casein kinase 1 (CK1) and glycogen synthase kinase 3β (GSK3β), and APC proteins. This intricate process involves the sequential phosphorylation of β-catenin by CK1 and GSK3β, leading to its recognition by the beta-transducin repeat-containing protein (β-TrCP) and subsequent ubiquitination and proteasomal degradation [19]. However, when the Wnt ligand binds to its receptor, the pathway gets activated, and a cascade of events is set in motion. The Wnt ligands interact with a seven-transmembrane receptor, Frizzled (F_Z_D), forming a larger cell surface complex together with lipoprotein receptor-related protein (LRP) 5/6 co-receptors. This complex activates the dishevelled (Dvl) proteins, which recruits the Axin complex to the receptors, leading to the displacement of GSK3β from the Axin complex. Consequently, β-catenin escapes degradation, accumulates in the cytoplasm, and translocates to the nucleus [19]. In the nucleus, β-catenin binds to LEF/TCF transcription factors, displacing co-repressors and recruiting co-activators. This intricate interplay ultimately activates the expression of the Wnt target genes, thereby influencing crucial cellular processes including cell proliferation, survival, and migration [19]. 

Given the essential roles played by WBC signaling in development and overall well-being, it is no wonder that mutations to the components of this pathway have been linked to several congenital disabilities, cancer development, and other serious diseases [25]. The strong correlation between aberrant WBC signaling and various cancer types is firmly established. This connection is exemplified by mutations affecting key components of the Wnt pathway, encompassing events like the silencing or inactivation of proteins of the Wnt secretory cascade or their co-factors [37,38,39]. Nevertheless, within the WBC signaling pathway, the prevalent mutation implicated in oncogenic progression pertains to alterations in the β-catenin gene [40]. Deregulation in the WBC signaling cascade is observed in the majority of tumorigenesis stages, from tumor development to metastasis and resistance to drugs [41]. Furthermore, modulation of this pathway may perturb cancer immune surveillance, enhancing the evasion of immunotherapies and hindering the effectiveness of immune checkpoint blockers [42,43,44]. Despite the advancements in human genome sequencing technologies and the characterization of the component proteins of major pathways, the role of Wnt signaling in cancer biology is intricate and complex, and its effects are not yet fully understood [45].

## 3. Wnt Signaling in HNC

The development of HNC consists of sequential alterations to the cellular and molecular pathways in the squamous epithelium, leading to gradual proliferation from pre-malignant lesions to tumors [46]. The WBC signaling pathway is crucial in the development of HNSCC, where the abnormal activation of the WBC signaling pathway has been found to contribute to the malignant transformation of cells, leading to tumor formation. The vital role of elevated WBC signaling in the initiation, development, and progression of HNSCC has been well documented in several studies. For instance, Yang and his colleagues elucidated that introducing a mutated β-catenin gene into HNSCC cells inhibited death receptor-mediated apoptosis and enhanced invasion of these tumor cells [47]. Another study revealed that abnormal accumulation of β-catenin in cytoplasm upregulated MMP-7 and induced epithelial-mesenchymal transition (EMT), ultimately resulting in the invasion and migration of oral squamous cell carcinoma (OSCC) cells [48]. The accumulated evidence has proven that, apart from invasion, lymph node metastasis (LNM) is a critical prognostic factor of HNSCC [49]. A recent study has proved that the WBC pathway promoted the invasion and LNM of HNSCC by partially activating Slug [50]. These studies demarcated that the dysfunctions of this pathway induced malignant transformation and metastasis of HNSCC; therefore, targeting WBC signaling may be a potential therapeutic approach in the treatment of this cancer.

## 4. Crosstalk between ncRNAs and WBC Signaling in HNSCC

The 21st century witnessed the discovery of transcripts that do not code for any proteins due to the successful achievements of the Human Genome Project and the subsequent initiative termed ENCODE (The Encyclopedia of DNA Elements project) in 2005 (ENCODE Project Consortium, 2012) [51]. The major classes of regulatory ncRNAs include miRNAs, piwi-interacting RNAs (piRNAs), long non-coding RNAs (lncRNAs), circular RNAs (circRNAs), and enhancer RNAs (eRNAs), along with housekeeping ncRNAs, including rRNAs, tRNAs, small nuclear RNAs (snRNAs), small nucleolar RNAs (snoRNAs), and telomerase RNAs [51]. miRNAs are small transcripts with an average length of 22 nucleotides that usually bind to 3′ UTR of mRNA and regulate its gene expression [52]. About 40% of miRNA genes are present in the intronic regions or within the exons of other genes from which they are transcribed into primary miRNAs (pri-miRNAs). Pri-miRNAs are processed by DROSHA into precursor miRNAs (pre-miRNAs) which are then transported to the cytoplasm by exportin 5 (XPO5). Furthermore, they are cleaved by DICER into small RNA duplexes which are then loaded onto the Argonaute (AGO) protein, which makes the miRNA single-stranded. Hence, the miRNA, along with the AGO and other co-factors, forms the miRNA-RNA-induced silencing complex (RISC) which is responsible for binding and inhibiting target mRNAs [53].

**Figure 1:**
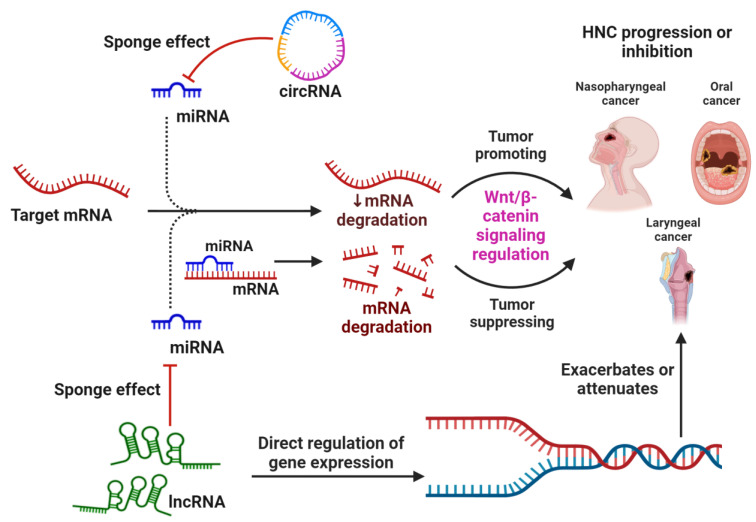
The general mechanism by which non-coding RNAs, specifically miRNAs, circRNAs, and lncRNAs, regulate the mRNA expression of genes associated with the WBC signaling pathway and thereby resulting in the suppression or progression of HNC types. Apart from directly regulating gene expression, circRNA and lncRNA also act by sponging miRNAs, which prevents their normal function of binding and inhibiting the target mRNA.

lncRNAs are a highly heterogenous group of transcripts that are more than 200 nucleotides in length and do not code for any protein [31,54]. They can be transcribed in the sense or anti-sense direction and they are called long intergenic RNAs (lincRNAs) when transcribed from intergenic regions [31]. Various categories of lncRNAs are transcribed from multiple DNA elements, including enhancers, promoters, and intergenic regions within eukaryotic genomes. Diverse mechanisms contributing to lncRNA biogenesis encompass processes such as RNaseP-mediated cleavage to form mature ends, the generation of snoRNA and the establishment of caps at their extremities through the assembly of protein snoRNP complexes, as well as the formation of circular structures. However, the exact processes involved in the synthesis and regulation of various lncRNAs remain unknown [55]. In contrast to miRNAs, lncRNAs employ a diverse array of mechanisms. They engage in interactions with transcriptional regulatory proteins, as well as bind complementarily to mRNAs or directly to miRNAs. The process by which lncRNAs sequester miRNAs is often referred to as the “sponge effect.” Through this mechanism, lncRNAs modulate the regulatory influence of miRNAs on gene expression (Figure 1) [31]. 

Another subclass of ncRNAs is represented by circRNAs, which are ubiquitously distributed in the blood, various bodily fluids, and tissues [56]. circRNAs are produced from pre-mRNA by back-splicing, where a 5′ splice site joins back to a 3′ splice site to form a closed head-to-tail continuous molecule, termed as circRNA [57]. Their inherent circular configuration provides circRNAs a remarkable stability, rendering them resilient to nuclease-mediated degradation [56]. Expression profiles of circRNAs display tissue and cell specificity, and these molecules play pivotal roles in developmental processes, cellular proliferation, innate immunity, neuronal functions, and the pathogenesis of an array of diseases, including malignancies [56]. 

A growing body of literature has demonstrated the importance of ncRNAs in human malignancies [58,59]. They have been found to act as either oncogenes or tumor suppressors, thereby influencing the development and progression of cancer [59,60]. Moreover, plenty of these ncRNAs can be discharged from cancer cells into bodily fluids, acting as diagnostic and prognostic markers [61]. Several ncRNAs have been found to modulate the proteins in the WBC pathway, which in turn regulates the tumorigenesis, invasion and migration, angiogenesis, and metastasis of HNSCC [62,63]. In addition, the modulation of the WBC pathway by ncRNAs, primarily miRNAs and lncRNAs, affects cancer cell proliferation, resistance to treatments, and poorer prognoses in HNC.

### 4.1. Interplay between ncRNAs and the WBC Pathway in Modulating Cell Proliferation and Survival

**Figure 2:**
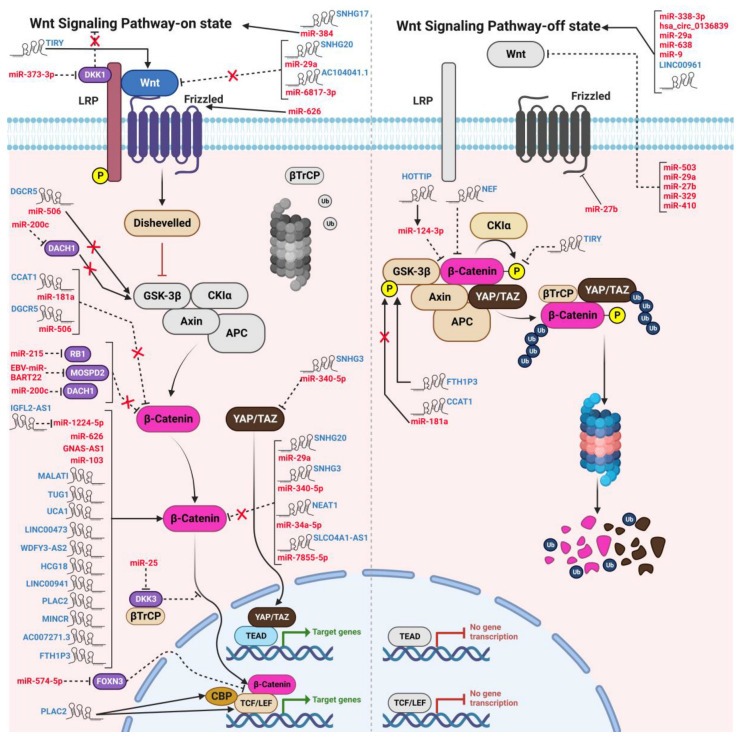
The modulatory functions of oncogenic and tumor suppressive ncRNAs on the “switch on” and “switch off” states of WBC signaling in HNCs. lncRNAs, circRNAs, and miRNAs bind to and induce or attenuate the expression of various components in the WBC pathway, thereby influencing the development of different types of HNCs. The mechanism of action of the WBC pathway, both in the ON-state and OFF-state, is also depicted in the figure.

Research indicates that anomalies in Wnt signaling can induce unbridled cellular proliferation, thereby fostering tumor initiation and advancement [19]. Specifically, in HNSCC cells, perturbed Wnt signaling has been linked with augmented cell proliferation and viability, concomitant with diminished apoptosis [64]. Furthermore, various ncRNAs have been found to either manifest oncogenic attributes or function as tumor suppressors through interactions with distinct constituents of the WBC signaling cascade. Such interactions either exacerbate or attenuate the severity of the malignancy, as delineated in Table 1 and Table 2, and presented in Figure 2 [60,65].

**Figure 3:**
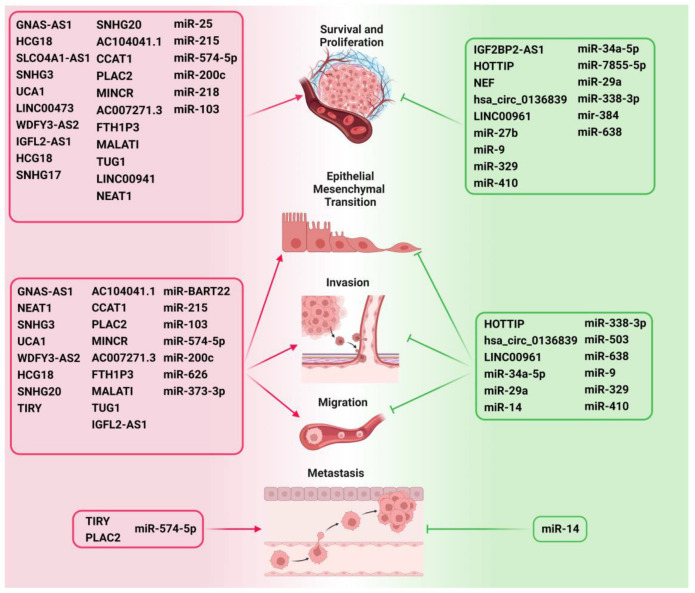
ncRNAs as critical modulators of various hallmarks of HNC such as survival, proliferation, EMT, invasion, migration, and metastasis by regulating Wnt/β-catenin signaling. The ncRNAs that promote the important hallmarks of cancer development and progression are depicted on the left side of the figure and are highlighted in red. The ncRNAs that inhibit cancer progression are depicted on the right side and are highlighted in green.

A comprehensive body of research exemplifies the involvement of lncRNAs and miRNAs in mediating cell proliferation in HNSCC cells through the modulation of the Wnt signaling pathway (Figure 3). For example, Mao et al. (2022) documented elevated levels of the lncRNA human leucocyte antigen complex group-18 (HCG18) in HNSCC cell lines and tissues. Amplification of HCG18 was found to potentiate HNSCC cell proliferation by interacting with cyclin D1, an integral protein in the Wnt cascade, while its suppression reduced proliferation. Moreover, HCG18-depleted cells exhibited marked downregulation in Axin2, c-Myc, survivin, and β-catenin [62]. Chen and colleagues (2019) illustrated that the activation of lncRNA placenta-specific protein 2 (PLAC2) via H3K27 acetylation induced OSCC cell proliferation, a phenomenon corroborated by the upregulation of cyclin D1 and Ki-67 levels [63]. Concurrently, a series of investigations consistently identified various lncRNAs, namely AC007271.3, MINCR, Taurine upregulated gene 1 (TUG1), and IGF2BP2-AS1, that augmented cell proliferation and viability in OSCC cells [66,67,68]. In a distinct study, Ai et al. (2020) showed that the activation of long intergenic non-protein coding RNA 941 (LINC00941) by EP300 (a histone acetyltransferase) in OSCC cells via H3K27 promoter modification led to increased CAPRIN2 expression through DNA looping, ultimately amplifying the canonical Wnt signaling pathway as evidenced by upregulated MYC, CCND1, SOX9, β-catenin, and p-LRP6 [69].

Furthermore, another study demonstrated that SLCO4A1-AS1 functions as a competing endogenous RNA (ceRNA) binding to miR-7855-p, leading to SETD7 upregulation. This lncRNA also upregulated the WBC pathway proteins, which augmented cell proliferation while inhibiting apoptosis [70]. Further investigations by Lin et al. (2023) and Zhao et al. (2023) revealed that lncRNA WDFY3-AS2 and IGFL2-AS1, respectively, induced cell proliferation in OSCC and tongue squamous cell carcinoma (TSCC) cells by influencing the WBC pathway [71,72]. Liang and team identified the role of lncRNA metastasis-associated lung adenocarcinoma transcript 1 (MALAT1) in enhancing tongue cancer cell proliferation through canonical Wnt signaling modulation [73]. Moreover, studies indicated that miRNAs such as miR-25 and miRNA-215 elevated cell proliferation and colony formation in nasopharyngeal cancer (NPC) by enhancing the expression of WBC pathway proteins [74,75]. Lastly, Sun et al. (2019) highlighted that overexpression of the lncRNA urothelial carcinoma associated 1 (UCA1) in LC cells increased β-catenin levels, promoting cell proliferation [76].

Several studies have reported the role of lncRNAs in modulating the WBC signaling pathway and its components by functioning as “miRNA sponges”. For instance, Jin et al. (2020) reported that overexpression of the lncRNA TIRY in cancer-associated fibroblasts (CAFs) activated the WBC signaling pathway, promoting the proliferation of OSCC cells. This upregulation was attributed to a sponging mechanism that reduced miR-14 expression [77]. Concurrently, Qiao et al. (2022) reported that lncRNA SNHG17 promoted oral cancer cell proliferation by acting as a decoy and inhibiting miR-384 [78]. In another study, lncRNA AC104041.1 was identified as a sponge for miR-6817-3p, resulting in the increased proliferation of OSCC cells [79]. Li et al. (2019) demonstrated that HCG18 exerted oncogenic effects in NPC cells by functioning as a ceRNA for miR-140, leading to the upregulation of both the WBC and Hedgehog signaling pathways [80]. Additionally, another study elucidated the oncogenic potential of lncRNA CCAT1 in enhancing OSCC cell proliferation and inhibiting apoptosis by activating the WBC pathway and suppressing miR-181a [81]. Chen et al. (2020) detected a marked upregulation of disheveled-Axin domain containing 1 (DIXDC1) and lncRNA small nucleolar RNA host gene 20 (SNHG20), paired with the targeted suppression of miR-29a in OSCC cells and tissues. This upregulation in SNHG20 promoted cell viability and proliferation while inhibiting apoptosis in OSCC cells, correlating with a poor prognosis for OSCC patients. Interestingly, treating cells with a miR-29a mimic considerably suppressed OSCC cell proliferation by downregulating Wnt-3a and β-catenin proteins. However, introducing DIXDC1 to cells treated with si-SNHG20 and miR-29a mimics intensified these effects, suggesting that SNHG20 facilitates OSCC progression via the miR-29a/DIXDC1/Wnt signaling axis [82]. Cao and Sun (2019) demonstrated that miR-200c enhanced proliferation and cell viability in NPC cells by upregulating canonical Wnt signaling proteins and suppressing the cell fate determinant factor, Dachshund family transcription factor 1 (DACH1) [83]. In another study, Xiong et al. (2020) identified that lncRNA HOTTIP facilitated the proliferation of TSCC cells by targeting miR-124-3p and influencing Wnt signaling [84]. Furthermore, Kang et al. (2020) elucidated that lncRNA SNHG3 augmented cell viability and glycolysis in LSCC cells by modulating the WBC pathway and targeting the miR-340-5p/YAP1 axis [85]. 

An emerging number of studies have investigated the role of ncRNAs in the suppression of proliferation and survival, and the induction of apoptosis of HNSCC cells by modulating the WBC signaling pathway. For instance, a recent study demonstrated that an siRNA-mediated knockdown of circRNA hsa_circ_0136839 markedly enhanced the cell cycle progression and cell proliferation of NPC cells by upregulating Wnt signaling proteins such as β-catenin and cyclin D1. However, this was significantly reduced by overexpressing this circRNA. Therefore, this study reinforced the notion that the aberrant expression of circRNA results in the development of HNSCC [56]. Additionally, numerous miRNAs, miR-9, miR-638, miR-329, miR-410, and miR-27b, were reported to repress the growth and proliferation of different OSCC cell lines by targeting key proteins involved in canonical Wnt signaling, such as frizzled_7_ (FZD7), Wnt-7b, phospholipase D1 (PLD1), and CXCR4 [86,87,88,89]. Furthermore, lncRNA LINC00961 suppressed the proliferation of TSCC by downregulating WBC signaling [90]. Furthermore, another study reported that miR-384 induced apoptosis and DNA fragmentation and inhibited the proliferation of LC cells via Wnt signaling. In addition, this study also proved that miR-384 specifically targeted and inhibited the expression of Wnt-induced secreted protein-1 (WISP1) gene [91]. Another study conducted in hypopharyngeal cancer established that miR-338-3p suppressed proliferation by targeted inhibition of metalloproteinase 17 or ADAM17, thereby subsequently downregulating β-catenin and cyclin D1 [92]. Likewise, lncRNA NEF also abolished cell proliferation and induced apoptosis in LC cells by downregulating WBC signaling [93].

Interestingly, a range of natural compounds has demonstrated anticancer properties against diverse cancer types, including HNC [10,94,95,96,97,98,99,100,101,102]. Importantly, a couple of studies have provided critical evidence regarding the potential of natural compounds to inhibit the progression of HNC by modulating the Wnt/β-catenin signaling pathway. For instance, Xiao and his group showed that curcumin, a compound extracted from turmeric (*Curcuma longa*) inhibited the proliferation of OSCC cells by upregulating miR-9 and repressing WBC signaling [103]. In addition, another study revealed that isoliquiritigenin, a flavonoid extracted from licorice root (*Glycyrrhizae radix*), suppressed NPC cell growth and proliferation as well as induced apoptosis by suppressing miR-32 and downregulating canonical Wnt signaling [104]. Therefore, it is imperative to study the effect of natural compounds in modulating the expression of ncRNAs through WBC signaling. Taken together, these studies suggest that ncRNAs are not only involved in regulating gene expression, but they are also able to orchestrate multiple cellular processes such as proliferation, survival, and viability by regulating WBC signaling to ultimately enable or suppress HNSCC progression.

**Table 1:** Wnt signaling modulating oncogenic ncRNAs in HNC.

ncRNA	Type of Study	Cell Line/Cancer Model	Target	Mechanism/Mode of Action	Reference
Nasopharyngeal Cancer					
EBV-miR-BART22 ^b^	In vitro	CNE1, CNE2, SUNE1 (Overexpression)	MOSPD2	↑Cell migration, invasion, N-cadherin, vimentin, Snail, β-catenin, EMT ↓E-cadherin, MOSPD2	[105]
EBV-miR-BART22 ^b^	In vitro	C666-1 (siRNA-mediated knockdown)	-	↑E-cadherin, MOSPD2 ↓Cell invasion, migration, N-cadherin, vimentin, Snail, β-catenin, EMT	[105]
EBV-miR-BART22 ^b^	In vivo	Hepatic metastasis BALB/c nude mice model (Overexpression)	-	↑Cell motility, tumor invasiveness	[105]
miR-25 ^b^	In vitro	HONE-1 (miRNA inhibitor)	DKK3	↑Apoptosis, DKK3 ↓Colony formation	[74]
miR-25 ^b^	In vitro	HONE-1 (Overexpression)	-	↑TCF4, c-Myc, Cyclin D1	[74]
miR-215 ^b^	In vitro	C666-1 (Overexpression)	RB1	↑Cell proliferation, migration, EMT, N-cadherin, vimentin, p-β-catenin ↓RB1, E-cadherin	[75]
miR-215 ^b^	In vitro	C666-1 (miRNA inhibitor)	RB1	↑RB1, E-cadherin ↓Cell proliferation, migration, p-β-catenin, N-cadherin, vimentin	[75]
miR-103 ^b^	In vitro	CNE1, SUNE1 (Overexpression)	TIMP3	↑β-catenin, CyclinD1, invasion, migration, proliferation ↓TIMP3	[106]
GNAS-AS1 ^a^	In vitro	SUNE1 (siRNA-mediated knockdown)	β-catenin	↓Cell proliferation, c-Myc, Cyclin D, MMP-2, β-catenin, invasion, migration	[107]
miR-574-5p ^b^	In vitro	C666-1 (Overexpression)	FOXN3	↑Cell viability, β-catenin, TCF4, invasion, metastasis ↓ FOXN3	[108]
HCG18 ^a^	In vitro	SUNE1, CNE2 (siRNA-mediated knockdown)	miR-140	↑miR-140, apoptosis, Caspase-3 and 9 ↓Cell growth, migration, invasion, Cyclin D1, β-catenin, c-Myc, Hedgehog signaling	[80]
miR-140 ^b^	In vitro	SUNE1, CNE2 (Overexpression)	HCG18	↓HCG18, Cyclin D1	[80]
miR-200c ^b^	In vitro	CNE2, SUNE1 (miR-200c-inhibitor)	DACH1	↑DACH1 ↓Cell proliferation, colony number, migration, β-catenin, c-Myc, GSK3β, Cyclin D1	[83]
NEAT1 ^a^	In vitro	CNE1, CNE2, SUNE1, SUNE2, 5-8F	miR-34a-5p	↓miR-34a-5p	[109]
NEAT1 ^a^	In vitro	5-8F (siRNA-mediated knockdown)	miR-34a-5p	↑ miR-34a-5p, E-cadherin ↓β-catenin, Cyclin D1, and c-Myc, N-cadherin, vimentin, cell proliferation, invasion, migration, EMT	[109]
NEAT1 ^a^	In vivo	SCID mouse xenografts (5-8F (shRNA mediated knockdown) xenografts)	miR-34a-5p	↑ miR-34a-5p, E-cadherin ↓Tumor growth, β-catenin, Cyclin D1, c-Myc, N-cadherin, vimentin	[109]
Laryngeal Cancer					
SLCO4A1-AS1 ^a^	In vitro	SNU46, TU177 (shRNA-mediated knockdown)	miR-7855-5p	↑miR-7855-5p ↓Cell proliferation, colony formation, β-catenin, Cyclin D1, c-Myc	[70]
SNHG3 ^a^	In vitro	TU177, AMC-HN-8 (shRNA-mediated knockdown)	-	↑Apoptosis, miR-340-5p, E-cadherin ↓Cell viability, glycolysis, YAP1, β-catenin, c-Myc, Bcl-2	[85]
SNHG3 ^a^	In vivo	BALB/c nude mice xenograft (shRNA-mediated knockdown)	-	↑miR-340-5p ↓Tumor volume, weight, YAP1	[85]
UCA1 ^a^	In vitro	AMC-HN-8 (Overexpression)	-	↑Cell proliferation, invasion, migration, β-catenin ↓ p-GSK3β	[76]
UCA1 ^a^	In vitro	AMC-HN-8 (siRNA-mediated knockdown)	-	↓Cell proliferation, invasion, migration	[76]
DGCR5 ^a^	In vitro	Hep2R	miR-506	↑DGCR5, ↓miR-506, CSC-like phenotype	[87]
DGCR5 ^a^	In vitro	Hep2R (siRNA-mediated knockdown)	miR-506	↑GSK3β, ↓Sox2, Oct4, Nanog, spheroid formation, β-catenin, Cyclin D1	[87]
DGCR5 ^a^	In vitro	Hep2R (siRNA-mediated knockdown and Radiation)	-	↓Radioresistance	[87]
miR-506 ^b^	In vitro	Hep2R (Overexpression)	-	↓Sox2, Oct4, Nanog, β-catenin, Cyclin D1	[87]
miR-506 ^b^	In vitro	Hep2R (Overexpression and Radiation)	-	↓Radioresistance	[87]
LINC00473 ^a^	In vitro	SCC25, CAL27 (shRNA-mediated knockdown)	-	↑Apoptosis, Bax, ↓Cell viability, colony number, Bcl-2, β-catenin, c-Myc	[65]
LINC00473 ^a^	In vitro	SCC9 (Overexpression)	-	↑ Cell viability, colony number, Bcl-2 ↓Bax, Apoptosis	[65]
LINC00473 ^a^	In vitro	SCC25, CAL27 (shRNA-mediated knockdown and radiation)	-	↑Apoptosis, Bax, ↓Cell viability, colony number, Bcl-2, β-catenin, c-Myc	[65]
Oral Cancer					
WDFY3-AS2 ^a^	In vitro	CAL27, SCC9 (siRNA-mediated knockdown)	-	↑E-cadherin ↓Cell proliferation, invasion, migration, vimentin, β-catenin, Myc, Slug	[72]
IGFL2-AS1 ^a^	In vitro	CAL-27, SCC-15, SCC-9, SCC-4 (shRNA-mediated knockdown)	miR-1224-5p	↑E-cadherin ↓Cell proliferation, invasion, migration, EMT, nuclear β-catenin, c-Myc, Cyclin D1, MMP-7	[71]
HCG18 ^a^	In vitro	HN30, SCC-4 (Overexpression)	-	↑Cell proliferation, migration, invasion, Cyclin D1	[62]
HCG18 ^a^	In vitro	HN30, SCC-4 (siRNA-mediated knockdown)	-	↓Cell invasion, migration, AXIN2, c-Myc, survivin, Cyclin D1, β-catenin	[62]
HCG18 ^a^	In vivo	Nude mice xenograft (Overexpression)	-	↑Tumor weight, volume	[62]
SNHG17 ^a^	In vitro	YD-38, SCC-9 (siRNA-mediated knockdown)	miR-384	↑Apoptosis ↓Cell proliferation, viability, CTNNB1, ELF1, Wnt/β-catenin signaling	[78]
miR-626 ^b^	In vitro	Ca9-22, HSC2 (miRNA inhibitor)	-	↑RASSF4, E-cadherin ↓vimentin, N-cadherin, invasion, migration, FZD1, β-catenin	[110]
miR-626 ^b^	In vitro	Ca9-22, HSC2 (Overexpression)	RASSF4	↑Invasion, migration, N-cadherin, β-catenin, FZD1 ↓E-cadherin	[110]
IGF2BP2-AS1 ^a^	In vitro	CAL27, SCC-9 (knockdown)	-	↑G1 phase arrest, apoptosis, Bax ↓Cell proliferation, colony formation, β-catenin, Cyclin D1, Bcl-2, MMP-2	[111]
LINC00941 ^a^	In vitro	HSC-3, OSC-19 (dCas9 tagged with KRAB-MeCP2)	-	↓Cell proliferation, colony formation, cell number, CAPRIN2, β-catenin, p-LRP6, MYC, CCND1, SOX9	[69]
LINC00941 ^a^	In vivo	Nude mice (HSC-3 xenograft dCas9 tagged with KRAB-MeCP2)	-	↓ Tumor formation, tumor weight	[69]
SNHG20 ^a^	In vitro	SCC-9 (siRNA-mediated knockdown)	miR-29a	↑Apoptosis, miR-29a ↓Cell viability, invasion, migration, Wnt-3a, β-catenin	[82]
miR-29a ^b^	In vitro	SCC-9 (Overexpression)	-	↓Cell viability, invasion, migration, Wnt-3a, β-catenin	[82]
miR-29a ^b^	In vitro	SCC-9 (miRNA inhibitor)	-	↑SNHG20	[82]
TIRY ^a^	In vitro	Oral CAFs (Overexpression)	-	↑Snail, Zeb1, α-SMA, β-catenin ↓miR-14	[77]
TIRY ^a^	In vitro	Tca8113 (CAF-conditioned media) (Overexpression)	-	↑Invasion, metastasis, Snail, Wnt-3a ↓Phosphorylation of β-catenin	[77]
TIRY ^a^	In vitro	Tca8113 (CAF-conditioned media) (siRNA-mediated knockdown)	-	↑miR-14	[77]
miR-14 ^a^	In vitro	Tca8113 (CAF-conditioned media) (Overexpression)	-	↓Invasion, metastasis	[77]
HOTTIP ^a^	In vitro	SCC25, UM1 (siRNA-mediated knockdown)	miR-124-3p	↑miR-124-3p, E-cadherin ↓Cell growth, invasion, migration, β-catenin, c-Myc	[84]
HOTTIP ^a^	In vivo	Nude mice (sh-HOTTIP OTSCC xenografts)	-	↑miR-124-3p, E-cadherin ↓Tumor weight, tumor volume, β-catenin, c-Myc, HMGA2	[84]
AC104041.1 ^a^	In vitro	SCC4 (shRNA-mediated knockdown)	miR-6817-3p	↓Cell viability, migration, Wnt-2b, β-catenin, c-Myc, vimentin	[79]
AC104041.1 ^a^	In vitro	CAL27 (Overexpression)	miR-6817-3p	↑Cell viability, migration, Wnt-2b	[79]
AC104041.1 ^a^	In vivo	BALB/c nude mice (SCC4 xenografts) (shRNA-mediated knockdown)	-	↓Tumor volume	[79]
AC104041.1 ^a^	In vivo	BALB/c nude mice (CAL27 xenografts) (Overexpression)	-	↑Tumor volume	[79]
CCAT1 ^a^	In vitro	KB, Cal-27 (shRNA-mediated knockdown)	miR-181a	↑Apoptosis, Bax, miR-181a, Caspase-3 and -9 ↓Cell proliferation, colony formation, Bcl-2, Cyclin D1, CDK4, invasion, migration, p-GSK3β, β-catenin and c-Myc	[81]
CCAT1 ^a^	In vivo	BALB/c mice with Cal-27 xenograft (shRNA-mediated knockdown)	-	↓Tumor size, weight, p-GSK-3β, β-catenin, c-Myc, Cyclin D1, Ki-67	[81]
PLAC2 ^a^	In vitro	SCC-9 (Overexpression)	-	↑Cell proliferation, Ki-67, invasion, migration, β-catenin, TCF-4, MMP-7 and -9, Cyclin D1	[63]
PLAC2 ^a^	In vitro	CAL-27 (siRNA-mediated knockdown)	-	↓Cell proliferation, Ki-67, Migration, Invasion, β-catenin, TCF-4, MMP-7 and -9, Cyclin D1	[63]
PLAC2 ^a^	In vivo	BALB/c nude mice (SCC-9 xenograft) (Overexpression)	-	↑Tumor volume, metastasis, PLAC2, CBP, β-catenin	[63]
MINCR ^a^	In vitro	SCC-25, TSCCA (shRNA-mediated knockdown)	-	↑Apoptosis, G0/G1 cell cycle arrest, Cleaved caspase-3 and -9, E-cadherin ↓Cell proliferation, migration, invasion, N-cadherin, β-catenin, c-Myc, Cyclin D1	[67]
AC007271.3 ^a^	In vitro	SCC-9, SCC-15 (siRNA-mediated knockdown)	-	↑Apoptosis ↓Cell proliferation, cell growth, Colony formation, invasion, migration, β-catenin, c-Myc, Cyclin D1, Bcl-2	[68]
AC007271.3 ^a^	In vitro	SCC-9, SCC-15 (Overexpression)	-	↑β-catenin, c-Myc, Cyclin D1, Bcl-2	[68]
AC007271.3 ^a^	In vivo	SCC-9 nude mice xenograft (Overexpression)	-	↑Keratinization, abnormal nuclear division, Ki-67, CD44, β-catenin, c-Myc, Cyclin D1, Bcl-2	[68]
FTH1P3 ^a^	In vitro	SCC-4, SCC-25 (siRNA-mediated knockdown)	-	↓Cell viability, invasion, β-catenin, p-AKT, p-GSK3β	[112]
miR-373-p ^b^	In vitro	SCC-9, UM1 (Overexpression)	DKK1	↑N-cadherin, vimentin, Cell invasion, viability, β-catenin ↓E-cadherin, CK18, DKK1	[113]
miR-373-3p ^b^	In vitro	SCC-9, UM1 (miRNA inhibitor)	-	↓N-cadherin, vimentin, invasion, cell viability, β-catenin	[113]
miR-218 ^b^	In vitro	UM1cis, Cal-27cis (anti-miR)	PPP2R5A	↑Cisplatin sensitivity, apoptosis, PPP2R5A ↓Cell viability, MRP1, ABCG2, p-gp, TopoIIβ, EZH2	[114]
miR-218 ^b^	In vitro	UM1cis (Overexpression)	PPP2R5A	↑ β-catenin, GSK3β, MRP1, ABCG2, p-gp, TopoIIβ, EZH2, Cell viability, cell growth ↓PPP2R5A	[114]
MALAT1 ^a^	In vitro	TSCC (shRNA-mediated knockdown)	-	↑E-cadherin, Bax, Apoptosis ↓Cell growth, invasion, migration, vimentin, β-catenin	[73]
MALAT1 ^a^	In vitro	TSCC (Overexpression)	-	↑Cell growth, invasion, migration, vimentin, β-catenin ↓E-cadherin, Bax, apoptosis	[73]
TUG1 ^a^	In vitro	Tca8113, TSCCA (siRNA-mediated knockdown)	-	↑Apoptosis, Caspase-3 activity, Cleaved caspase-3 and -9, Bax ↓Cell proliferation, growth, colony formation, invasion, Bcl-2, β-catenin, c-Myc, Cyclin D1	[66]

^a^: long non-coding RNA; ^b^: micro RNA; ↑ Upregulation; ↓ Downregulation.

**Table 2:** Wnt signaling modulating tumor-suppressive ncRNAs in HNC.

ncRNA	Type of Study	Cell Line/Cancer Model	Target	Mechanism/Mode of Action	Reference
Hypopharyngeal Cancer					
miR-503 ^b^	In vitro	FaDu (Overexpression)	-	↓Cell invasion, WNT-3A, BCL11B, and CCND2, MMP-3, -7, and -9, FGF7, CTGF	[60]
miR-338-3p ^b^	In vitro	FaDu (Overexpression)	ADAM17	↓Cell proliferation, ADAM17, cell migration, invasion, cyclin D1, MMP-2, nuclear β-catenin, p-pRb, Wnt/β-catenin	[92]
miR-338-3p ^b^	In vitro	FaDu (Inhibitor)	-	↑β-catenin, cyclin D1, p-pRb, MMP-2, sox-2, Nanog	[92]
Laryngeal Cancer					
miR-384 ^b^	In vitro	TU212, TU686	WISP1	↑Cell apoptosis, DNA fragmentation, Caspase-3 ↓Cell proliferation, WISP-1	[91]
miR-384 ^b^	In vitro	TU212, TU686 (Inhibitor)	-	↓Caspase-3, DNA fragmentation	[91]
NEF ^a^	In vitro	UM-SCC-17A (Overexpression)	-	↑Cell apoptosis ↓Cell proliferation, β-catenin	[93]
Nasopharyngeal Cancer					
hsa_circ_0136839 ^c^	In vitro	CNE2 (Overexpression)	-	↓Cell proliferation, invasion, migration colony formation, G0/G1 cell cycle arrest, β-catenin	[56]
hsa_circ_0136839 ^c^	In vitro	C666-1 (siRNA-mediated knockdown)	-	↑Cell proliferation, invasion, migration colony formation, β-catenin, c-Jun, LEF1, CD44, cyclin D1	[56]
Oral Cancer					
miR-503 ^b^	In vitro	SAS, OECM1	-	↓Cell invasion, WNT-3A, BCL11B, CCND2, MMP-3, 7, and 9, FGF7, CTGF	[60]
miR-638 ^b^	In vitro	SCC-9 (Overexpression)	PLD1	↓Cell proliferation, invasion, migration, PLD1, β-catenin, c-Myc, Cyclin D1	[115]
miR-638 ^b^	In vitro	SCC-9 (Inhibitor)	PLD1	↑PLD1, β-catenin, c-Myc, Cyclin D1	[115]
LINC00961 ^a^	In vitro	SCC-1 (Overexpression)	-	↑E-cadherin ↓Cell proliferation, invasion, migration, vimentin, N-cadherin, Snail, β-catenin	[90]
LINC00961 ^a^	In vitro	SCC-1 (shRNA-mediated knockdown)	-	↑Cell proliferation, Wnt/β-catenin signaling	[90]
miR-27b ^b^	In vitro	Tca8113, SCC-4 (Overexpression)	FZD7	↓Cell proliferation, FZD7, Wnt, Cyclin D1, c-Myc	[88]
miR-9 ^b^	In vitro	Tca8113, SCC-9 (Overexpression)	CXCR4	↑Cell apoptosis, G1/S cell cycle arrest ↓Cell proliferation, colony formation, cell invasion, CXCR4, β-catenin, Bcl-2, c-Myc	[86]
miR-9 ^b^	In vitro	Nude mice xenograft (Overexpression)	CXCR4	↓Tumor growth, CXCR4, Ki-67	[86]
miR-329^b^/miR-410 ^b^	In vitro	OEC-M1, SCC-15 (Overexpression)	Wnt-7b	↓Wnt-7b, TCF/LEF1transcriptional activity, cell proliferation, invasion, colony formation, β-catenin, p-GSK3β, c-Myc, Cyclin D1	[89]
miR-329^b^/miR-410 ^b^	In vitro	OC-3, SCC-4 (miR329-inhibitor/ miR410-inhibitor)	Wnt-7b	↑Wnt-7b, TCF/LEF1transcriptional activity, β-catenin, c-Myc, Cyclin D1	[89]
miR-329^b^/miR-410 ^b^	In vivo	OEC-M1 xenograft (overexpression of miR329/miR410)	Wnt-7b	↓Tumor weight, volume, Wnt-7b, β-catenin	[89]

^a^: long non-coding RNA; ^b^: micro RNA; ^c^: circular RNA; ↑ Upregulation; ↓ Downregulation.

### 4.2. Interplay between ncRNAs and the WBC Pathway in the Modulating EMT, Invasion, and Migration

EMT is a biological process characterized by the transformation of epithelial cells into a more mobile mesenchymal phenotype, allowing them to invade and migrate to new tissues [116]. Importantly, EMT, invasion, and migration represent interconnected biological phenomena that collectively contribute to the progression of tumors from a benign to a more aggressive malignant state [117]. These sequential events are orchestrated through a complex network of signaling pathways, initiated by the downregulation of cell adhesion molecule E-cadherin, mediated by proteins such as Snail, Slug, Zeb 1/2, smad interacting protein 1 (SIP1), or Twist 1. Simultaneously, there is an upregulation of vimentin and N-cadherin expression, facilitating the necessary alterations in cellular properties for efficient tissue invasion and migration [117,118,119].

Numerous investigations have elucidated the pivotal role of ncRNAs in modulating Wnt signaling, which in turn influences the invasive phenotype, indicating their central function in directing the invasion and migration of HNSCC cells (Figure 2). For instance, Wang et al. (2020) delineated that the lncRNA GNAS-AS1 activated the Wnt signaling pathway by upregulating β-catenin. Additionally, the suppression of this lncRNA markedly decreased the metastatic capacity of NPC cells by attenuating matrix metalloproteinase-2 (MMP-2) [107]. Another study documented that the Epstein-Barr virus-encoded miRNA BART-22 enhanced EMT, invasion, and migration of NPC cells, by directly targeting motile sperm domain-containing protein 2 (MOSPD2). This miRNA exerts its effect by modulating the Wnt signaling cascade [105]. Zhao et al. (2020) found that miR-103 significantly suppressed the tissue inhibitor of metalloproteinases-3 (TIMP-3) while enhancing β-catenin and cyclin D1 expressions, which intensified the invasive and migratory propensities of NPC cells [106]. Another investigation by Liu et al. (2018) revealed that lncRNA ferritin heavy chain 1 pseudogene 3 (FTH1P3) increased migration and invasion in OSCC cells. Moreover, FTH1P3 silencing critically downregulated the PI3K/Akt/GSK3β/WBC signaling cascade, as evidenced by reduced levels of β-catenin, phosphorylated Akt, and GSK3β [112]. Further studies have indicated that miR-574-5p enhanced invasiveness and migration in NPC cells by inhibiting the tumor suppressor gene forkhead box N3 (FOXN3), while miR-373-3p markedly aggravated EMT-induced metastasis by curbing DKK1, a negative regulator of Wnt signaling, in TSCC cells [108,113]. Additionally, miR-626 promoted EMT, invasion, and migration in OSCC cells through RASSF4 targeting and the consequent β-catenin signaling upregulation [110]. A notable finding by Chen et al. (2020) emphasized the lncRNA role of SNHG20 in amplifying OSCC cell migration and invasion by upregulating crucial WBC signaling elements β-catenin and Wnt-3a [82]. Concurrent studies have affirmed that lncRNAs like AC007271.3, MINCR, TUG1, IGF2BP2-AS1, and PLAC2, activated via H3K27 acetylation, escalate the invasion and migration in OSCC cells by modulating the canonical Wnt signaling pathway, thus advancing oral cancer progression [63,66,67,68,111]. Additionally, Lin et al. (2023) highlighted the pro-invasive effects of lncRNA WDFY3-AS2 by upregulating key proteins, including vimentin, slug, β-catenin, and c-Myc. Cao and Sun (2019) expounded on miR-200c’s crucial influence on augmenting NPC cell migration via direct DACH1 targeting [83]. Another investigation indicated that lncRNA MALAT1 promotes EMT and inhibits apoptosis in tongue cancer cells through the WBC signaling modulation [73].

Furthermore, another study demonstrated that the lncRNA TIRY activated the WBC signaling cascade within CAFs. This activation promoted EMT, invasion, and metastasis in OSCC cells by diminishing miR-14 levels through a sponging mechanism. At a mechanistic level, TIRY was observed to amplify the expression of molecular markers including Snail, Zeb1, α-SMA, Wnt-3a, and β-catenin [77]. Ji et al. (2019) reported the role of the nuclear paraspeckle assembly transcript 1 (NEAT1) in augmenting EMT, migration, and invasion of NPC cells by inhibiting miR-34a-5p directly. Intriguingly, silencing NEAT1 led to an upregulation of miR-34a-5p and a concomitant downregulation of β-catenin, cyclin D1, c-Myc, N-cadherin, and vimentin in both NPC cell lines and the SCID mouse xenograft model [109]. Additionally, another study highlighted that silencing lncRNA HCG18 attenuated invasion and metastasis in NPC cells by downregulating Hedgehog and WBC signaling pathways, as well as by sponging miR-140 [80]. Another study by Xiong et al. (2020) emphasized that the lncRNA HOTTIP enhanced invasion and migration in tongue cancer cells by targeted repression of miR-124-3p. Notably, silencing HOTTIP resulted in the upregulation of miR-124-3p which, in turn, attenuated invasion, migration, and tumor proliferation by targeting HMGA2 through Wnt signaling modulation [84].

A myriad of investigations has elucidated the pivotal role of ncRNAs in hampering EMT, invasion, migration, and metastasis in HNSCC. For instance, the tumor suppressor miRNA, miR-503, was shown to curtail HNSCC cell invasion by downregulating WBC pathway constituents, including Wnt-3a and MMPs 3, 7, and 9. This miRNA also decreased the expression of invasion-related genes, including fibroblast growth factor 7 (FGF7) and connective tissue growth factor (CTGF) [60]. Additionally, studies have confirmed that multiple miRNAs, such as miR-9, miR-638, miR-329, and miR-410, substantially reduced the invasive and migratory characteristics of OSCC cells by attenuating the WBC signaling cascade [86,89,115]. In another notable study, lncRNA LINC00961 was found to diminish invasion, migration, and EMT in TSCC cells, acting as a putative tumor suppressor in tongue cancer development [90]. This collective body of research emphasizes the integral role of ncRNAs in modulating the migration and invasion of cancer cells, making them essential focal points in oncological research.

### 4.3. Interplay between ncRNAs and the WBC Pathway in Modulating Chemoresistance and Radioresistance

Chemotherapy and radiotherapy constitute the cornerstones of therapeutic intervention for individuals afflicted with advanced or metastatic stages of HNSCC, as substantiated by pertinent research findings [120,121]. Cisplatin-based chemotherapy and concomitant radiotherapy or radio-chemotherapy are widely used against several HNSCC conditions, especially in unresectable tumors [121,122,123]. Nonetheless, in numerous instances, cancer cells develop resistance to these therapeutic modalities through a variety of mechanisms. One of these mechanisms involves the anomalous activation of the WBC signaling pathway, resulting in the upregulation of genes associated with chemoresistance. Notably, this includes genes involved in drug efflux pathways, such as ATP-Binding Cassette (ABC) transporters, as well as genes implicated in epigenetic regulations, such as DNA methyltransferases [124,125]. Several ncRNAs have been substantiated as effective regulators of chemoresistance and radio-resistance in HNSCC cells by modulating WBC pathway. For instance, an investigation demonstrated that miR-218 directly targets protein phosphatase 2 regulatory subunit B’alpha (PPP2R5A), a tumor suppressor gene, influencing cancer progression and chemoresistance in oral cancer cells. The study further indicated that when miR-218 is downregulated, there is an inhibition of Wnt signaling, enhancing the sensitivity of oral cancer cells to cisplatin-based treatments. Additionally, the repression of miR-218 led to the decreased expression of several genes implicated in chemoresistance, such as ABCG2, multidrug resistance protein 1 (MRP1), and p-glycoprotein (p-gp) [114]. Another study showed that the upregulation of chemotherapy-induced lncRNA 1 (CILA1) significantly enhanced chemoresistance in TSCC cells accompanied by elevated invasion, metastasis, and EMT in vitro and in vivo. This study also confirmed that CILA1 exerted its functions by the upregulation of the WBC signaling pathway [126]. 

Additionally, a couple of studies have also demonstrated the modulation of radioresistance in HNSCC cells by lncRNAs via WBC signaling. For instance, the lncRNA LINC00473 enhanced the radioresistance of LC cells by activating WBC signaling. This study also demonstrated that the silencing of LINC00473 and X-ray treatment induced apoptosis and suppressed colony formation and the proliferation of LC cells [65]. In addition, another study demonstrated that the lncRNA DGCR5 promoted radioresistance in LC cells by sponging miR-506. Further, the silencing of DGCR5 enhanced radiosensitivity and downregulated Wnt signaling components, including β-catenin and Cyclin D1 [87]. Hence, a deeper investigation into the WBC signaling pathway elucidating the regulatory roles of ncRNAs within this pathway and comprehending the downstream implications of its activation is essential. Such insights are crucial for devising novel therapeutic strategies to enhance the efficacy of both chemotherapy and radiotherapy and more effectively target HNC.

## 5. Conclusions

HNC encompasses a spectrum of malignant conditions affecting the oral, nasal, nasopharyngeal, hypopharyngeal, and laryngeal regions. Deviations in the expression of proteins involved in various cellular activities and pathways constitute a significant factor in the advancement of HNSCC. The WBC signaling pathway exemplifies the complex cellular communication systems vital to both developmental and pathological contexts. While the WBC pathway is fundamental for cellular proliferation, morphogenesis, and homeostatic balance, its aberrations can lead to severe consequences. Genetic alterations within this pathway are associated with diverse cancers, including HNC. Over recent years, ncRNAs have gained prominence as potential therapeutic targets in numerous cancers, including HNC. Emerging research underscores the instrumental role of ncRNAs in HNC, modulating various signaling pathways, including WBC. Consequently, ncRNAs impact key oncogenic processes in HNC, such as cellular proliferation, differentiation, invasion, EMT, migration, and metastasis. Clinical trials are increasingly evaluating the utility of several ncRNAs as diagnostic and therapeutic indicators in oncology. For instance, one noteworthy study highlighted the potential of MALAT1 targeting miR-124 as a diagnostic biomarker for OSCC, as denoted in trial NCT05708209. The investigation encompassed 20 OSCC patients and 20 healthy controls, assessing MALAT1 and miR-124 levels in unstimulated saliva samples. Furthermore, ncRNAs critically influence the chemotherapeutic and radiotherapeutic resistance of HNSCC cells. Given the importance of ncRNAs in HNC, a thorough understanding of their specific roles is vital for the innovation of refined therapeutic strategies. This review describes the central role of ncRNAs in modulating cancer cell behavior, particularly through the WBC signaling mechanism. Nonetheless, further studies are essential to thoroughly discern the contributions of ncRNAs in HNC progression and to design groundbreaking therapeutic interventions for this malignancy.

## Abbreviations

α-SMA	Alpha smooth muscle actin
β-TrCP	Beta-transducin repeat-containing protein
ABC	ATP-binding cassette
ADAM17	ADAM metallopeptidase domain 17
AKT	Protein kinase B
APC	Adenomatous polyposis coli
Bax	Bcl-2-associated X protein
BCL11B	B-cell lymphoma/leukemia 11B
Bcl-2	B-cell leukemia/lymphoma 2 protein
CD44	Cluster of differentiation 44
CILA1	Chemotherapy-induced long non-coding RNA 1
CK1	Casein kinase 1
c-Myc	Cellular myelocytomatosis oncogene
CTGF	Connective tissue growth factor
CXCR4	CXC chemokine receptor 4
DACH1	Dachshund family transcription factor 1
DGCR5	DiGeorge syndrome critical region gene 5
DKK1	Dickkopf-1
DKK3	Dickkopf Wnt signaling pathway inhibitor 3
ELF1	E74 Like ETS Transcription Factor 1
EZH2	Enhancer of zeste homolog 2
FGF7	Fibroblast growth factor 7
FZD7	Frizzled 7
GSK3β	Glycogen synthase kinase-3 beta
HCG18	HLA complex group 18
HMGA2	High mobility group A2
HNC	Head and neck cancer
HNSCC	Head and neck squamous cell carcinoma
HPV	Human papilloma virus
LC	Laryngeal cancer
LEF1	Lymphoid enhancer binding factor 1
LRP	Lipoprotein receptor-related protein
LNEC	Laryngeal neuroendocrine carcinoma
LNM	Lymph node metastasis
MALAT1	Metastasis Associated Lung Adenocarcinoma Transcript 1
MMP	Matrix metalloproteinase
MOSPD2	Motile sperm domain containing 2
MRP1	Multidrug resistance-associated protein 1
ncRNA	Non-coding RNA
NUAK1	NUAK family SNF1-like kinase 1
p-gp	P-glycoprotein
PLAC2	Placenta-specific protein 2
PLD1	Phospholipase D1
PPP2R5A	Protein phosphatase 2 regulatory subunit B’alpha
RASSF4	Ras association domain family member 4
SETD7	SET domain-containing 7 histone lysine methyl transferase
SLCO4A1	AS1-solute carrier organic anion transporter family member 4A1 antisense RNA 1
snoRNA	Small nucleolar RNA
SNHG1	Small nucleolar RNA host gene 17
SNHG3	Small nucleolar RNA host gene 3
TCF/LEF	T-cell factor/lymphoid enhancer factor 1
TCF4	Transcription factor 4
TIMP-3	Tissue inhibitor of metalloproteinases-3
TopoII	topoisomerase IIβ
TSCC	Tongue squamous cell carcinoma
TUG1	Taurine upregulated gene 1
UCA1	Urothelial Carcinoma Associated 1
WBC	Wnt/β-catenin
WIF1	Wnt inhibitory factor-1
WISP1	Wnt-induced secreted protein-1
YAP/TAZ	Yes-associated protein 1/transcriptional coactivator with PDZ-binding motif
